# Supplementary figures and images for: Thrombocytopenia in murine schistosomiasis is associated with platelet uptake by liver macrophages that have a distinct activation phenotype
Source: PLoS Pathog. 2025 Nov 26;21(11):e1013732. doi: 10.1371/journal.ppat.1013732 (PMC12697973; doi:10.1371/journal.ppat.1013732)

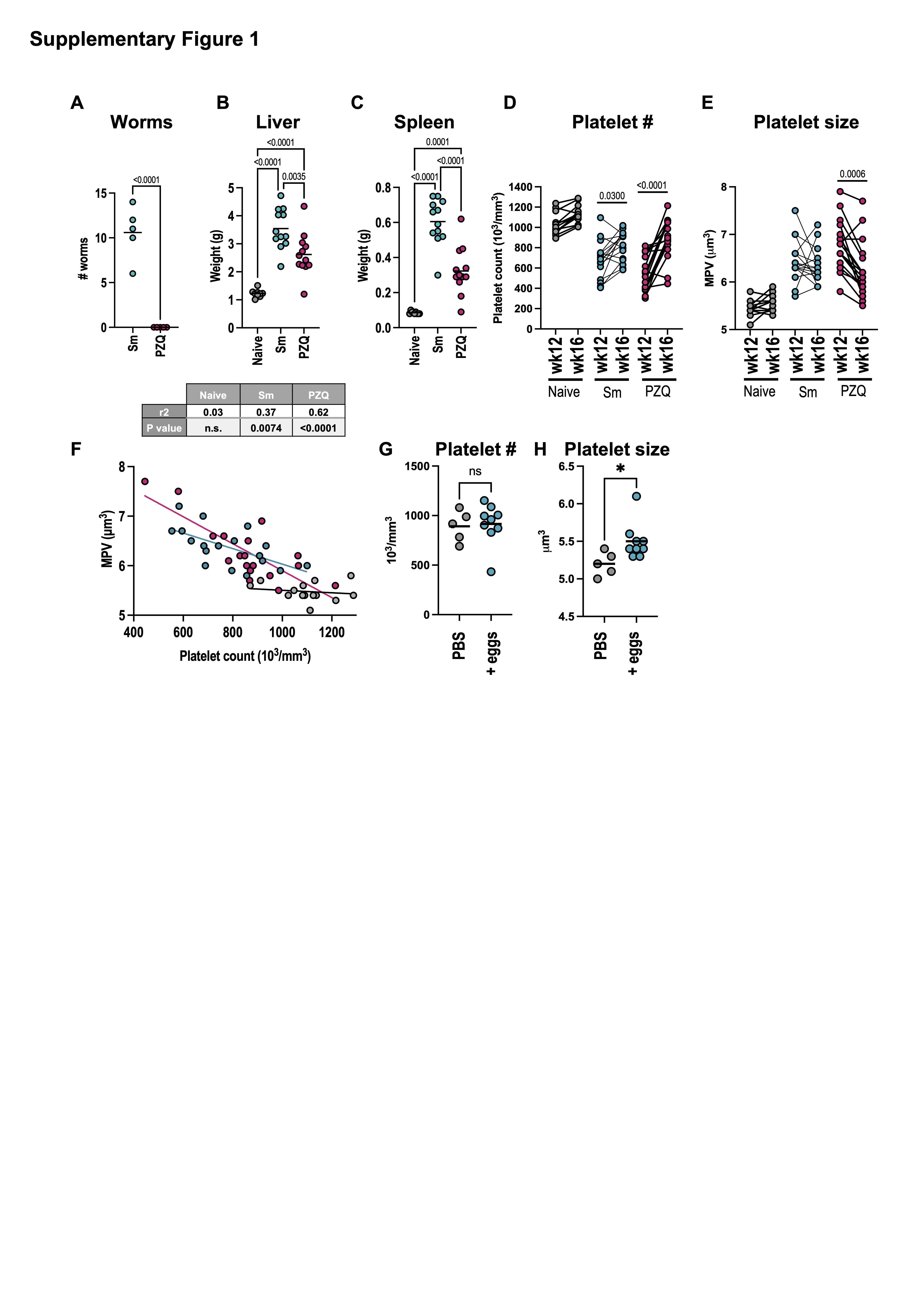

Supplement: S1 Fig — C57BL/6 mice were infected with 40–50 S. mansoni cercariae and treated -/ + PZQ at week 12 post-infection and 4 weeks later (A) Adult worms counts, (B) Liver and (C) Spleen weight at week 16 post-infection. (D-E) Pairwise comparison of (D) Platelet count and (E) MPV in individual animals at weeks 12 (pre-PZQ) and 16 (post-PZQ) post-infection in naïve, infected (Sm) and infected then PZQ-treated mice. (F) Correlation of platelet count and MPV in naïve, infected (Sm) and infected-cured (PZQ mice) at wk16. (G-H) Day 21 platelet number and MPV in mice injected with schistosome eggs on d0 (intraperitoneal) and d14 (intravenous). Data in B-H is pooled from 2-3 experiments with n = 2–6 per group. Significance determined by unpaired t-test (A, G-H), ANOVA with Tukey post-hoc test (B-C), paired t-test (D-E) or linear regression (F). (TIFF) [file ppat.1013732.s001.tiff]

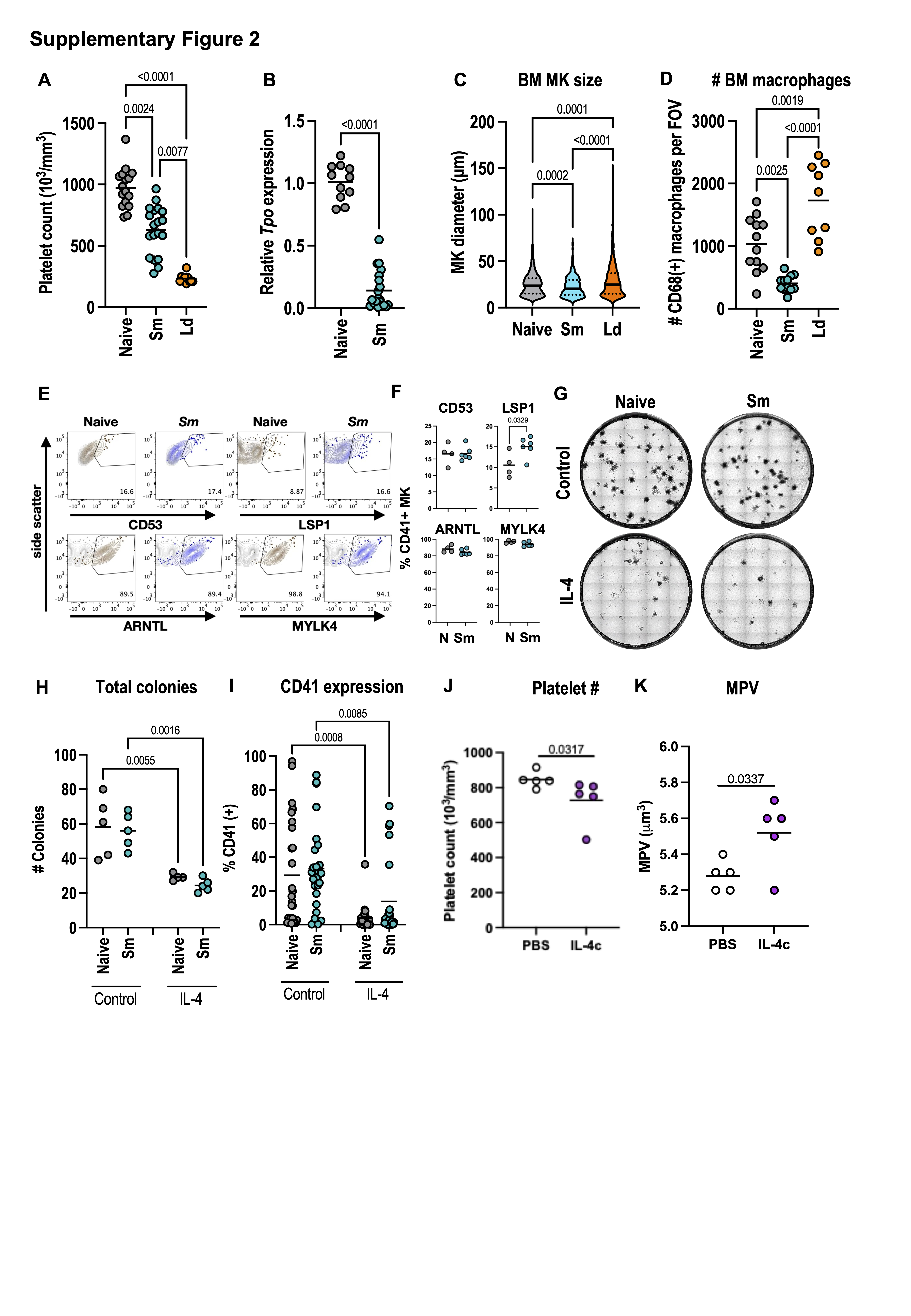

Supplement: S2 Fig — C57BL/6 mice were infected or not with S. mansoni (Sm) for 10 weeks or L. donovani (Ld) for 4 weeks and (A) platelet counts, (B) qPCR liver Thpo expression (C) BM MK diameter and (D) BM CD68+ macrophage cell number per field of view (FOV) determined. (E-F) Bone marrow MK expression of CD53, LSP-1, ARNTL and MYLK4 in naïve and schistosome infected mice determined by flow cytometry. FMO control stains are shown in light grey. (G) Representative colony formation assays using BM HSC (Lin- c-Kit+ EPCR+ Sca1+ CD48- CD150+) from naive and 12 week schistosome-infected mice -/ + exogenous IL-4. (H) Total colony count and (I) CD41+ cells within each colony are shown. (J) Platelet counts and (K) MPV in mice treated with IL-4c or PBS control as detailed in Materials and Methods. Data are pooled (A-D, H), representative (G, I) of 2–3 experiments, or from a single experiment (J-K). Significance is determined by ANOVA with Tukey post-hoc test, Kruskal-Wallis with Dunn’s test or unpaired t-test. (TIFF) [file ppat.1013732.s002.tiff]

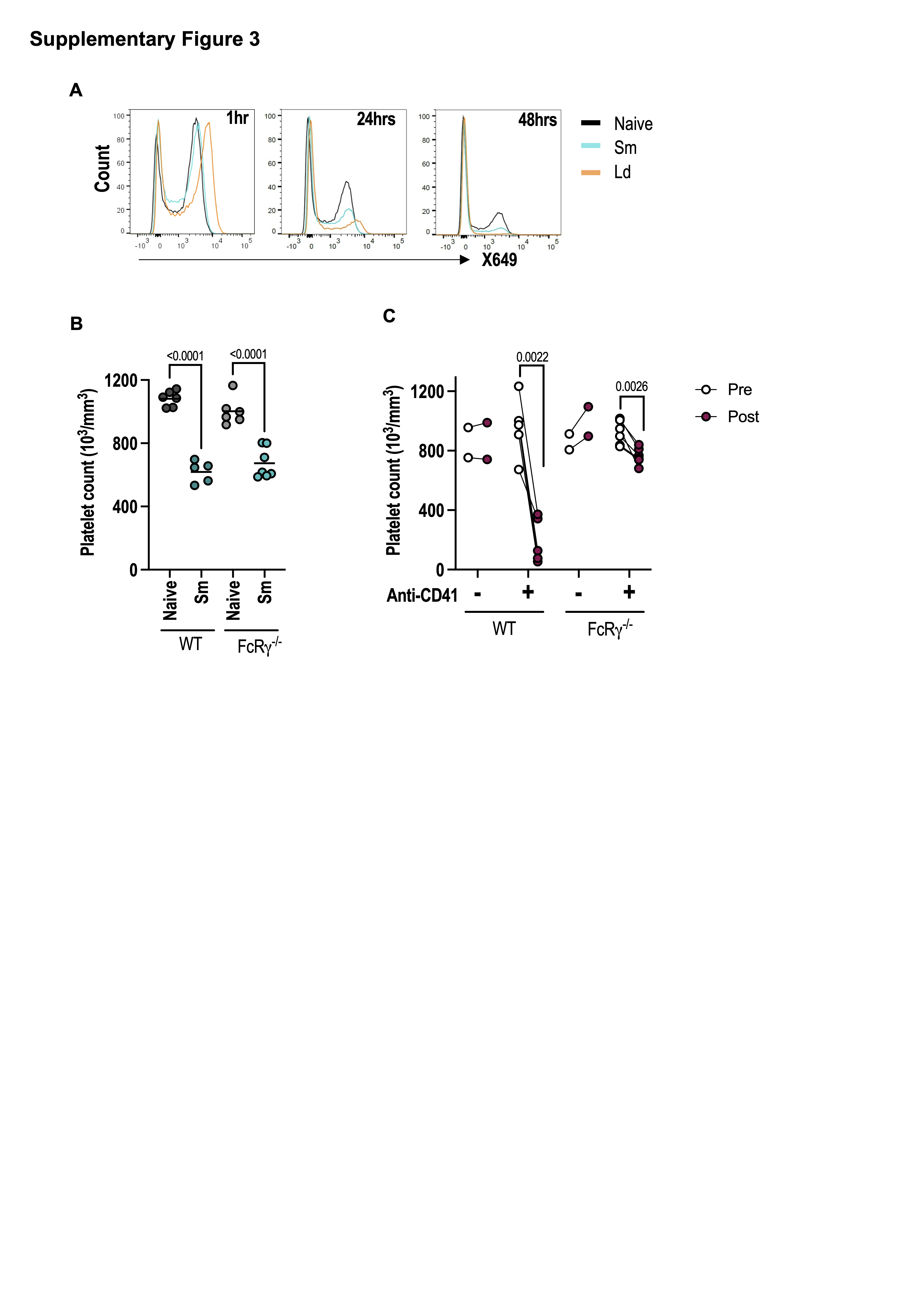

Supplement: S3 Fig — (A) Representative flow cytometry histograms showing X649 labelling of gated CD41+ platelets for naïve (black), 12 wk S. mansoni (blue) and 4wk L. donovani (orange)-infected mice at 1, 24 and 48 hours post-X649 treatment. (B) Platelet counts in WT and FcRγ-/- mice that are either naïve or 12 wk S. mansoni infection. (C) Platelet counts in WT and FcRγ-/- mice pre and 24 hours post-treatment with isotype control (-) or a depleting anti-CD41 mAb (+). Data representative of 2–3 experiments (A-B) or are a single experiment (C). Significance is determined by ANOVA with Tukey post-hoc test (B) or paired t-test comparing individual mice (C). (TIFF) [file ppat.1013732.s003.tiff]

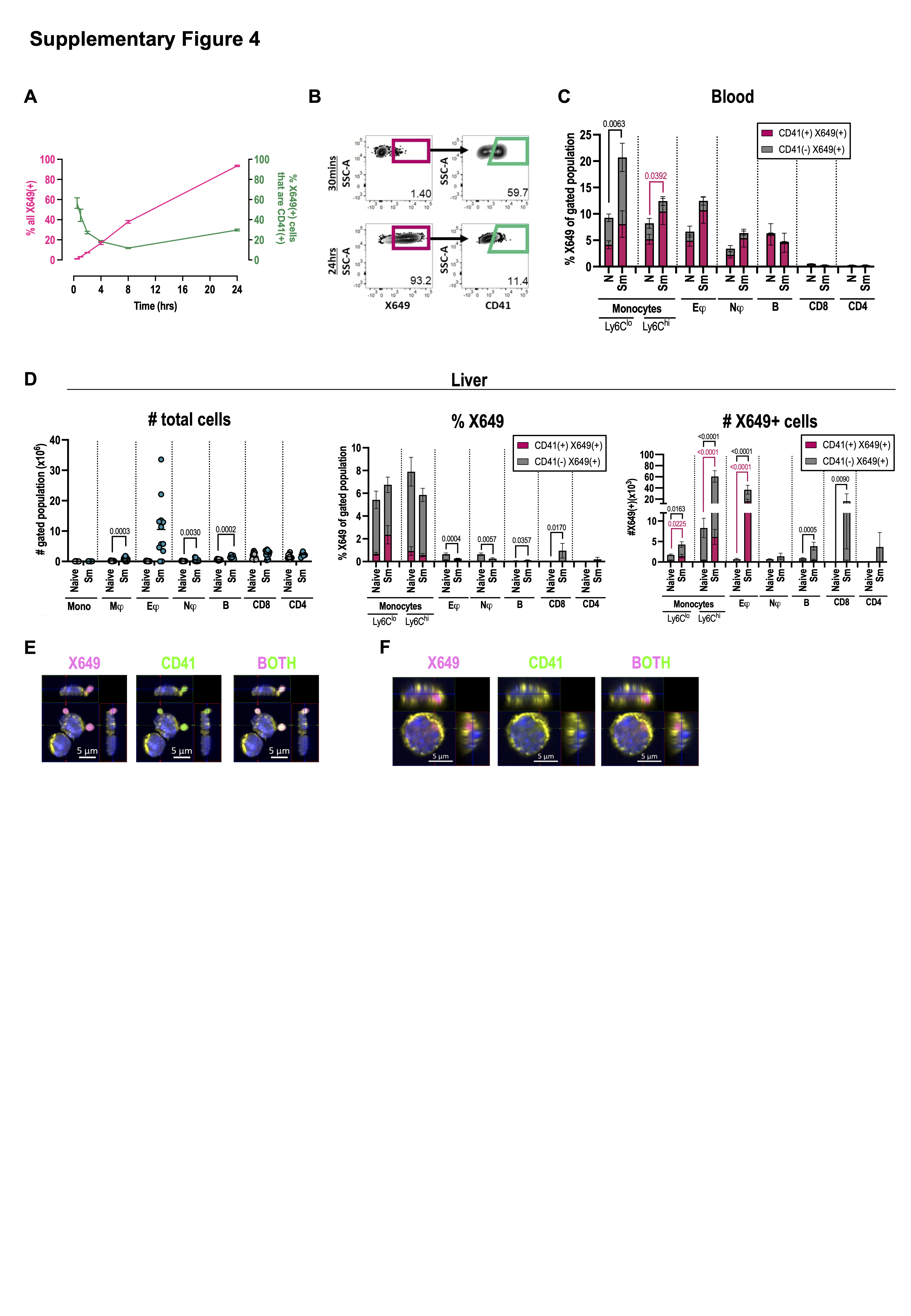

Supplement: S4 Fig — (A) BM-Mφ were co-cultured with X649-labelled platelets from naïve mice. Macrophages were recovered 0.5, 1, 2, 4, 8 and 24 hours later and stained for CD11b, CD64 and CD41. Proportion of macrophages (CD11b+ CD64+) that are X649+ shown in pink (left axis) and proportion of X649+ macrophages that have CD41+ surface stain shown in green (right axis). (B) Representative flow cytometry of macrophages following X649+ platelet co-culture for 0.5 and 24 hours with X649+ (pink) and surface CD41+ (green) indicated. Data in A-B from single experiment with 6 technical replicates at every time point, bars represents mean + /- SEM. (C) Naïve or S. mansoni infected (10–12 weeks) C57BL/6 mice were injected with anti-GPIb-V-IX conjugated DyLight 649 (X649) 48hrs prior to harvest. Blood X649+ leukocytes that are either CD41+ (pink) or CD41- (grey) in the blood are quantified and shown as proportion of each cell population. Monocytes are gated as CD11b+ CD115+ SiglecF- Ly6G- Ly6C+/-, eosinophils are CD11b+ SiglecF+ SShi, neutrophils as CD11b+ Ly6G+ SiglecF- SShi, B cells as CD19+ TCRb-, CD8 T cells as TCRb+ CD8a+, and CD4 T cells as TCRb+ CD4+. (D) As C for liver immune cells showing absolute number of different immune cells per liver, % of each cell type that is X649+, and number of each cell type that is X649+. Individual channels from Fig. 4G showing E) cell surface and F) internalised platelets associated with macrophage. Data in C-D pooled from 3 experiments and significance determined by unpaired t-test with comparisons between stacked bars colour coded. (TIFF) [file ppat.1013732.s004.tiff]

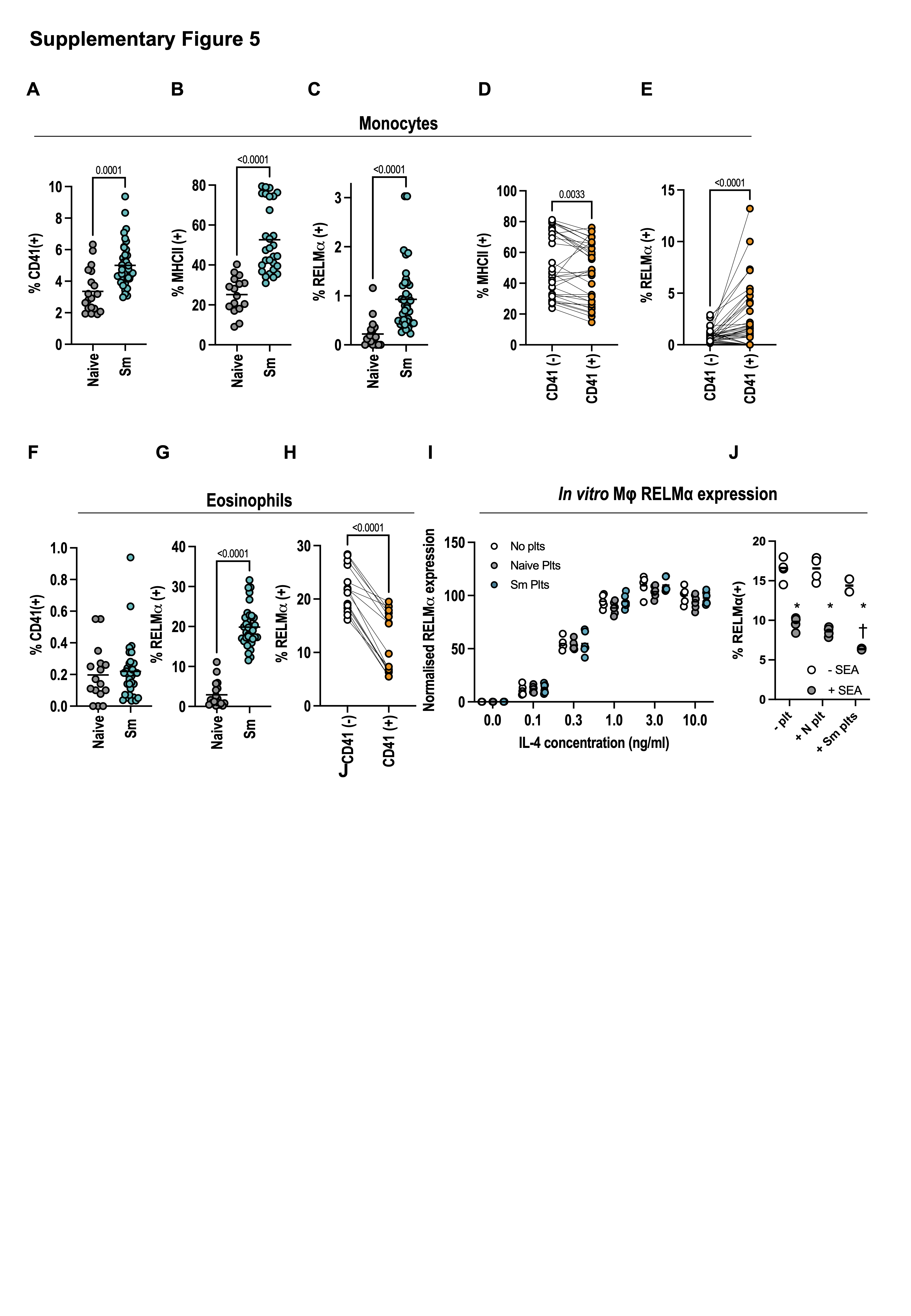

Supplement: S5 Fig — (A) CD41, (B) MHC II and (C) RELMα expression by liver monocytes (gated CD11b+ CD64+ SiglecF- Ly6G- Ly6C+ cells) from naïve C57BL/6 mice or following infection with S. mansoni (10–12 weeks). Data pooled from 4 experiments. Pairwise comparison of (D) MHC II and (E) RELMα expression by CD41- (white) vs CD41+ (orange) liver monocytes from infected mice with data pooled from 3 experiments. (F) CD41 and (G) RELMα expression by liver eosinophils (gated CD11b+ SiglecF+ SShi) as A-C. (H) Pairwise comparison of RELMα expression by CD41- (white) vs CD41+ (orange) eosinophils from infected mice. (I) BMMφ were cultured with indicated concentrations of IL-4, either alone (white) or with platelets from naïve (grey) mice or mice infected for 12wks with S. mansoni and RELMα expression determined 18hrs later by flow cytometry. Data pooled from 2 experiments and expression is normalised to 10ng/ml IL-4 (no platelets) group. (J) BMMφ were cultured with (grey) or without (white) SEA for 18hrs in the presence of 10ng/ml IL-4, either in the absence of platelets or with platelets from naïve or schistosome-infected mice. Significance in A-C and F-G determined by unpaired t-test, (D-E, H) by paired t-test between individual animals, and (J) by two-way ANOVA (* = p < 0.001 vs no SEA, † = p < 0.05 vs no platelets). (TIFF) [file ppat.1013732.s005.tiff]

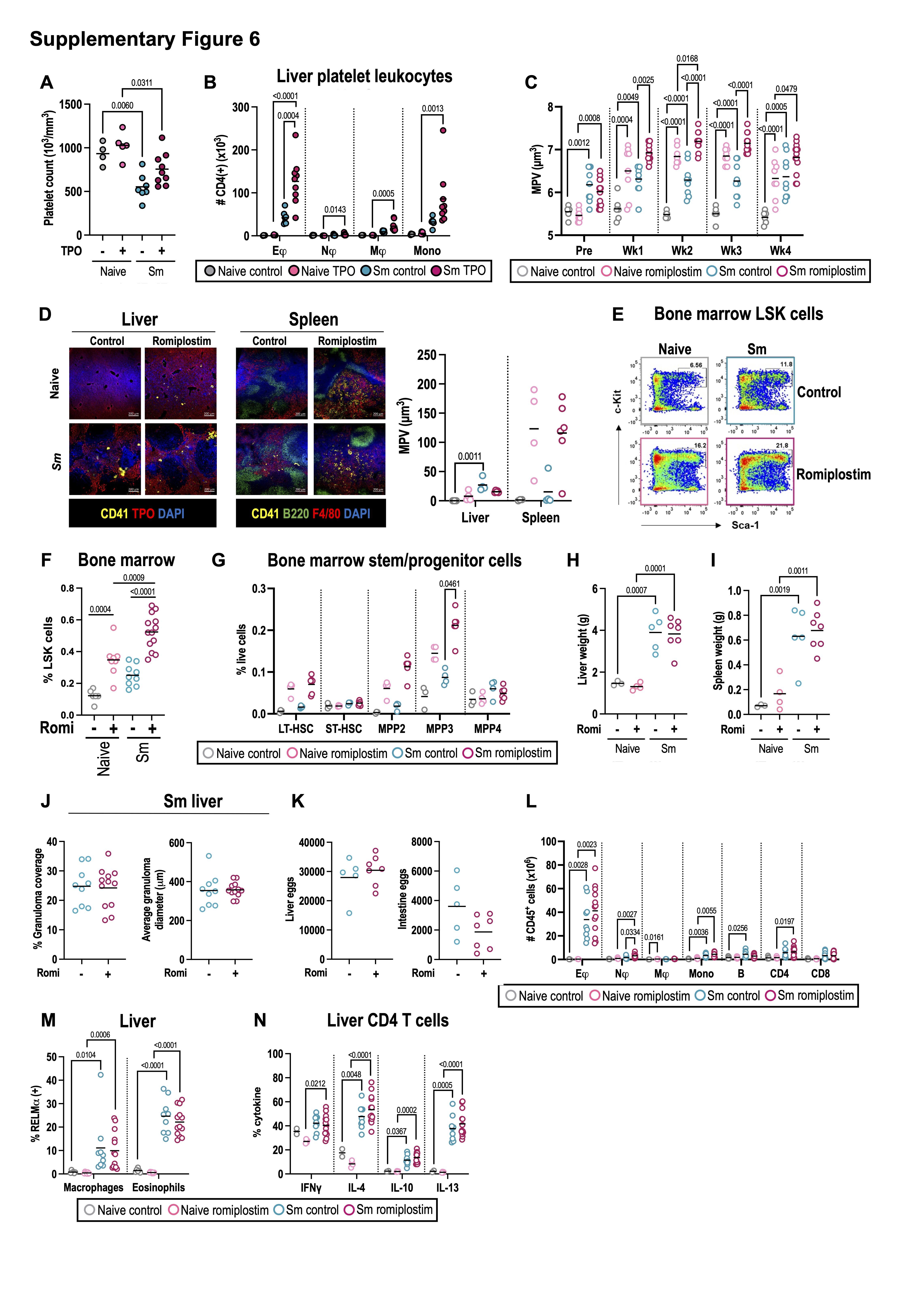

Supplement: S6 Fig — C57BL/6 mice -/ + S. mansoni were treated with human TPO 3x per week at week 8 and 9 post-infection with (A) blood platelets and (B) liver CD41 + platelet-leukocyte aggregrates (PLA) determined at wk10. (C) Platelet MPV in mice treated with romiplostim as Fig. 6A. (D) Representative liver (left) and spleen (right) sections to identify MK (nucleated CD41+ cells) with MK quantified per field of view (FOV). Livers stained for CD41 (yellow), TPO (red, hepatocytes), DAPI (blue, nuclei). Spleens stained for CD41 (yellow), B220 (green, B cells), F4/80 (red, macrophages), DAPI (blue, nuceli). (E) Representative flow cytometry BM LSK cells (lineage- Sca1 + c-kit+) and (F) % LSK of live BM cells in control (ctrl) and romiplostim (R) treated naive and infected (Sm) mice. (G) BM LSK subsets (LT-HSC = CD135- CD150+ CD48-; ST-HSC = CD135- CD150- CD48-; MPP2 = CD135- CD150+ CD48+; MPP3 = CD135- CD150- CD48+; MPP4 = CD135+ CD150- CD48+). (H-I) Liver and spleen weights in control and romiplostim-treated mice -/ + S. mansoni infection. (J) Quantification of granuloma coverage (left) and granuloma diameter (right) in livers from control or romiplostim-treated mice. (K) liver (left) and intestine (right) parasite eggs. (L) Number of the liver CD41+ leukocytes. (M) Percentage RELM⍺+ macrophages and eosinophils. (N) Percentage of liver CD4 T cells that are positive for the indicated intracellular cytokine following ex vivo stimulation. Data pooled or representative of 2 experiments, significance determined by unpaired t-test (J-K) or ANOVA with Tukey post-hoc test (other panels). (TIFF) [file ppat.1013732.s006.tiff]

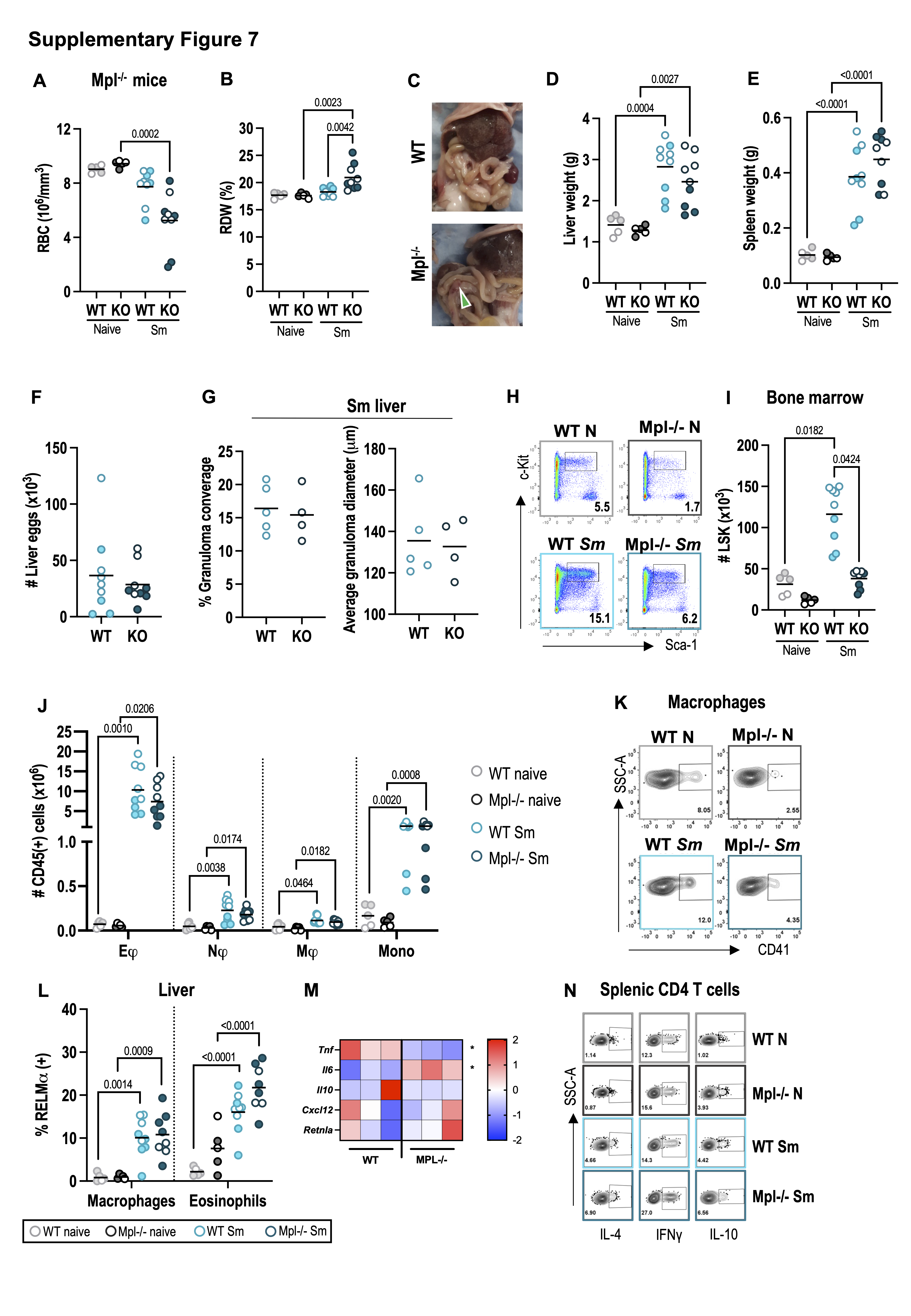

Supplement: S7 Fig — (A) Red blood cell count and (B) Red Density Width (RDW) in naive and S. mansoni-infected WT and Mpl-/- mice. Mice were infected with a lower dose on cercariae (30) and harvest at an earlier point (7.5wks) than other experiments to avoid host death. (C) Intestinal bleeding (arrow) in infected Mpl-/- mice, whereas liver pathology was seen in both WT and Mpl-/-. (D) Liver and (E) spleen weight in naïve and S. mansoni-infected WT and Mpl-/- mice. (F) Liver eggs (G) liver granuloma coverage and size of single egg granulomas in WT and Mpl-/- mice. (H) Representative flow cytometry of BM LSK cells (lineage- Sca1 + c-kit+) and (I) numbers of BM LSK in naive and infected WT and Mpl-/- mice. (J) Numbers of liver eosinophils (Eφ), neutrophils (Nφ), macrophages (Mφ) and monocytes (mono). (K) Representative CD41 flow cytometry staining for liver macrophages. (L) Liver macrophage and eosinophil RELMα determined by flow cytometry. (M) Z-scores of indicated transcripts in liver macrophages from infected WT and Mpl-/- mice determined by qPCR (N) Representative spleen CD4 T cells intracellular cytokine staining. Data for (A-F, H-L) pooled from two experiments and between male and female mice (open symbols male, closed symbols female). Significance determined by ANOVA with Tukey post-hoc test or unpaired t-test. (TIFF) [file ppat.1013732.s007.tiff]

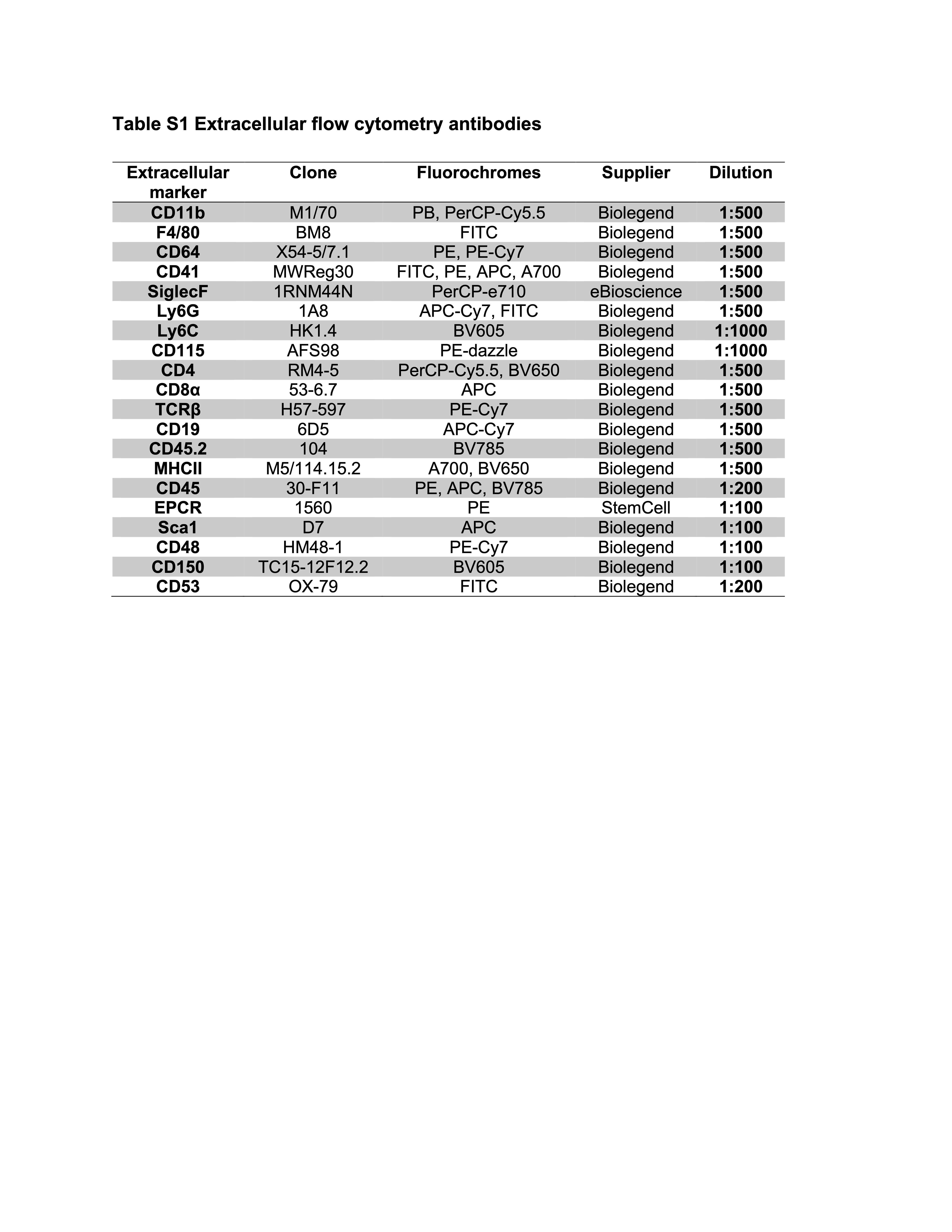

Supplement: S1 Table — (TIFF) [file ppat.1013732.s008.tiff]

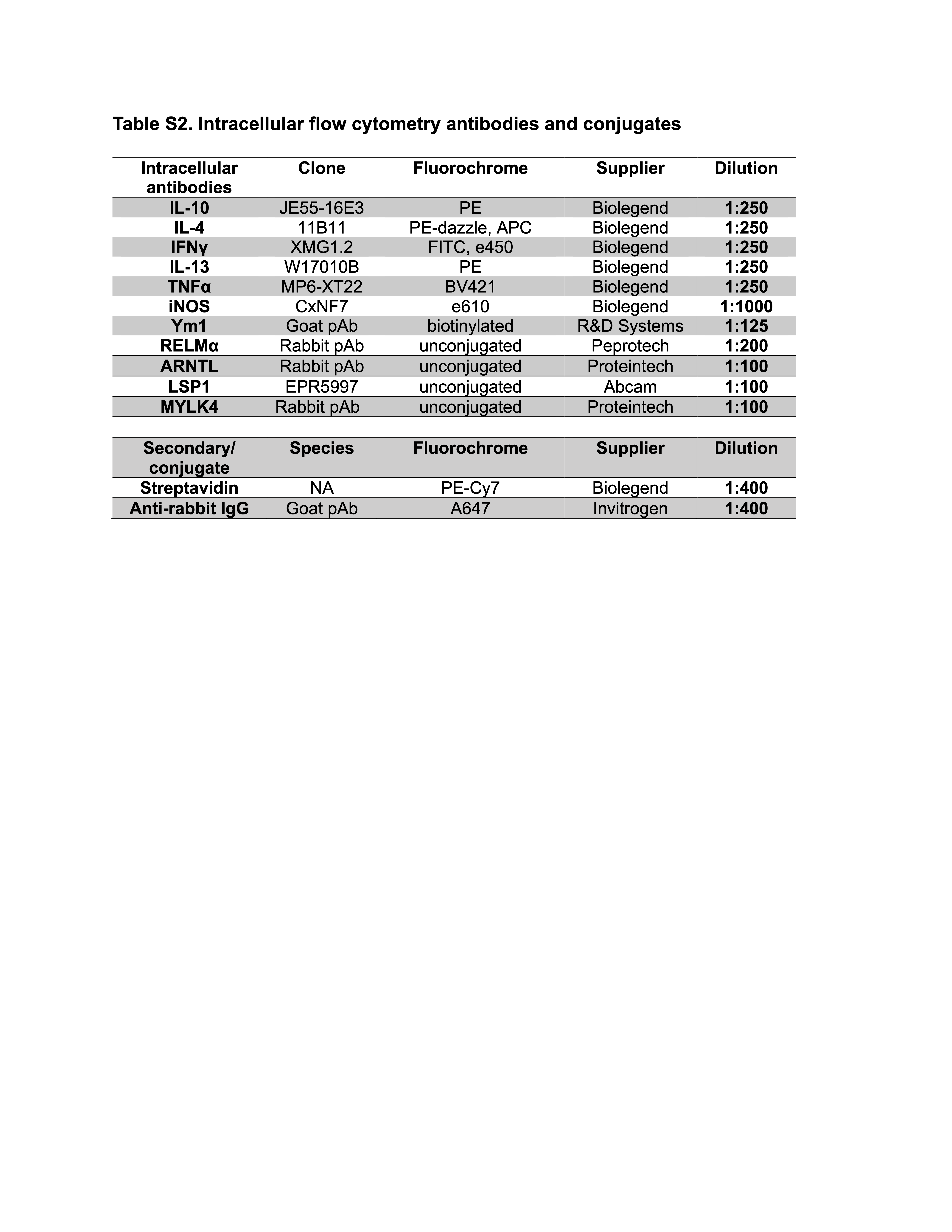

Supplement: S2 Table — (TIFF) [file ppat.1013732.s009.tiff]

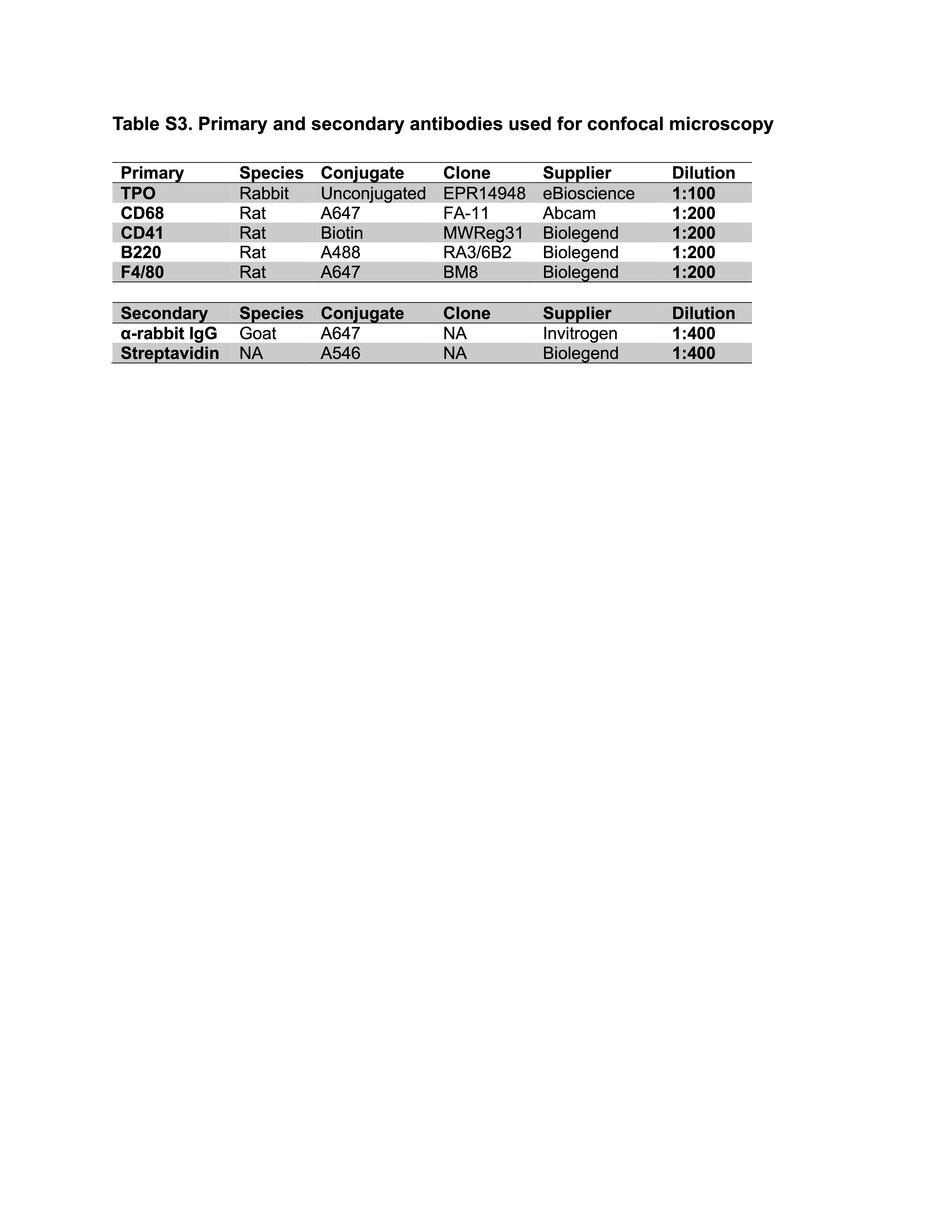

Supplement: S3 Table — (TIFF) [file ppat.1013732.s010.tiff]

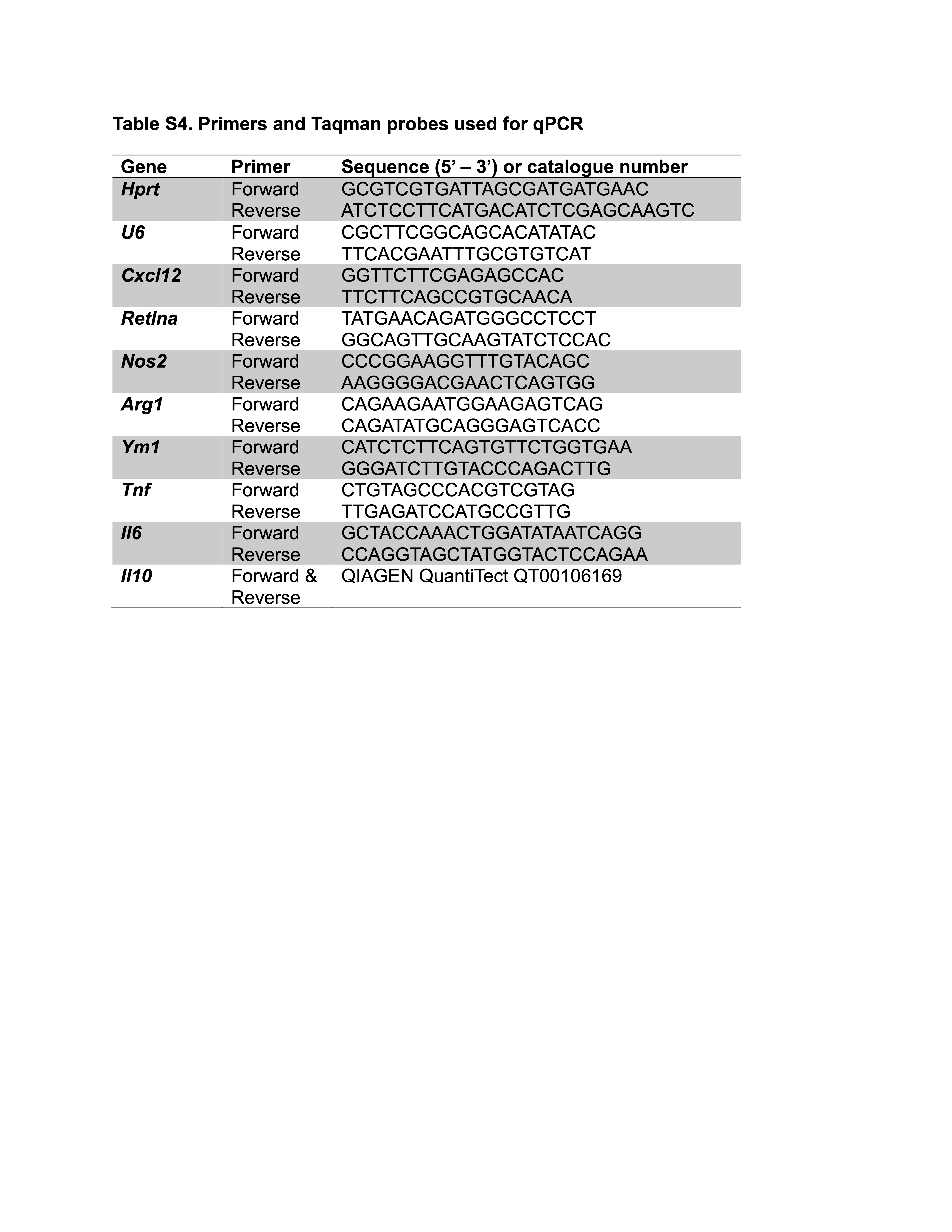

Supplement: S4 Table — (TIFF) [file ppat.1013732.s011.tiff]
