# Supplementary material for: Thrombocytopenia in murine schistosomiasis is associated with platelet uptake by liver macrophages that have a distinct activation phenotype
Source: PLoS Pathog. 2025 Nov 26;21(11):e1013732. doi: 10.1371/journal.ppat.1013732 (PMC12697973; doi:10.1371/journal.ppat.1013732)
Supplement: Attachment — Submitted filename: Greenman PLoS path_RESPONSE TO REVIEWERS.pdf [file ppat.1013732.s014.pdf]

York Biomedical Research Institute
Department of Biology
University of York
York YO10 5DD
+441904-328576
james.hewitson@york.ac.uk

9th July 2025

Dear PLOS Pathogens Editors

We are pleased to resubmit our paper "***Thrombocytopenia in murine schistosomiasis is associated with platelet uptake by liver macrophages that have a distinct activation phenotype***" for publication in *PLOS Pathogens*. We thank the Reviewers for their positive comments, and as detailed below, have carried out multiple additional experiments we think address their concerns.

Point by Point Response to Reviewers (our responses in blue):

Part I - Summary

Reviewer #1: In their manuscript "Thrombocytopenia in murine schistosomiasis is associated with platelet uptake by liver macrophages that have a distinct activation phenotype", Greenman and colleagues investigate the role of platelets on macrophage phenotype in schistosome infections. The study provides insights into the role of platelet in macrophage alterations following schistosoma infection. The study is potentially interesting, however, there are some points that need to be clarified.

Reviewer #2: This is an interesting and novel work which has investigated thrombocytopenia in schistosomiasis. The authors have used an impressive variety of murine intervention models to assess the role of platelets and their interaction with the immune system in schistosomiasis. The work is clearly written, the figures are clear and the science seems well-executed. Key findings are:

- Schistosomiasis causes thrombocytopenia which is maintained after PZQ treatment
- The causes of the thrombocytopenia are investigated: TPO is reduced in schistosomiasis, although MKs actually increase. This seeming contradiction may be explained by a change in ploidy of the MKs (to a less platelet-producing form). In addition, platelet clearance rate is increased. The increased rate of platelet clearance may be explained by increased binding and internalization of platelets by monocytes and macrophages.
- Platelet-bound macrophages had a different phenotype, although cause and effect is not conclusively determined.
- Increasing platelets (with romiplostin) does not seem to affect immune responses. Decreasing platelets (with anti-CD41 or MPL-/- mice) increases morbidity but also alters immune responses.

A key weakness of the study is that the authors have shown that macrophages associated with platelets have a different phenotype, but it is not clear if this can be attributed to the platelets. E.g. does binding platelets change the macrophage phenotype, or does having a different phenotype allow them to better bind platelets. However I am satisfied that the authors have discussed the difficulties of unpicking this in the final paragraph of the discussion.

Reviewer #3: Overall this is an intriguing study that demonstrates that schistosome infection modulates the lifecycle of platelets and that platelet myeloid interactions occurs during infection. This data fits well with recent work in the field that has demonstrated that schistosome infection modulates different facets of hematopoiesis. While the data presented is of a high quality, there are some lapses and missed opportunities in experimental design that make some of the conclusions in the discussion not well supported. Below are suggestions for data to add that would better support the conclusions around the potential role of platelets in modulating the immune response. The discussion should also better incorporate the entirety of work about parasite infections that modulate different facets of hematopoiesis as there are multiple papers published since 2021 that demonstrate alterations to various lineages.

We thank the reviewers for their supportive comments. In response to Reviewer 3's point about parasite infection and haematopoiesis, we did cite Wijshake et al 2024 in the Results section [52] and now also include this in the Discussion to show schistosome infection activates HSPC. In addition, we now also cite Francisco et al [42] in Results as they have shown MK present in the liver of schistosome-infected mice. Finally, in the Discussion, we include recent evidence that helminth infection induces innate immune training of both HSPCs and myeloid progenitors [65, 66].

Part II – Major Issues: Key Experiments Required for Acceptance

Reviewer #1:

1) The authors observe an increase in platelet size measured as MPV upon infection. I recommend to confirm that these platelets with increased size are young platelets by Thiazole staining, checking glycoprotein expression and also MHC-I expression.

We extensively characterised infection-induced changes to platelet phenotype in a parallel study that was recently published (Greenman et al, Exp Haem 2025) [79]. In that work, we used an unbiased proteomic approach to compare platelets from naive and schistosome-infected mice and found MHC-I is indeed one of the most significantly upregulated proteins in Sm-platelets. Using flow cytometry, we also showed larger platelets have higher levels of MHC-I compared to smaller platelets. Whilst we readily detected platelet glycoproteins GPIb α/β, GPIIb, GPIIIa, GPV and GPIX in our proteomics, their levels were unchanged after infection. Our current submission here is complementary yet distinct from [79] which identifies infection-induced changes to platelet phenotype, whereas this submission focuses on the mechanisms of thrombocytopenia and immune consequences. We have amended our discussion to reference the Exp Haem paper as it is now published.

2) Could the authors provide unstained control sections for the red TPO staining in Figure 2A as the liver tissue is highly autofluorescent. Did the authors also quantify TPO levels in liver lysate?

We now include a representative isotype control for TPO staining in Fig. 2A. We did not quantify TPO protein levels in liver homogenates, only serum samples. We did however measure *Thpo* message in naive and infected livers and now include this (new Fig. S2B).

3) Since there is an increased number of MKs in the liver following infection: Do you also see MK precursors and HSCs in the liver?

The impact of chronic helminth infection on the function of HSCs and other progenitors is a major research focus in our lab. Recently, Wijshake et al 2024 [52] showed HSC and MK precursors (MEP) are present in the liver of infected mice. The authors used an acute model of schistosome infection whereby mice are infected with a high dose of parasites (200 cercariae) for 7 weeks. In our experience, these infection levels cause extreme pathology and mouse death between weeks 7-9 post-infection. As such, we use a much lower parasite dose (30-50 cercariae), which allows mice to survive into the chronic stages of infection (as occurs in human disease). To look for liver HSC, we compared LSK cells (= lineage$^-$ Sca1$^+$ c-kit$^+$) in the bone marrow and liver of naive and 10wk schistosome infection mice using HSC reporter animals (Fgd5-Zsgreen; Gazit et al JEM 2014 PMID: 24958848). Our flow cytometry data (**Response Fig. 1**) shows infection induces a marked expansion of bone marrow LSK cells (as Wijshake et al 2024, and also Fig. S6E in our submission) and that BM Fgd5$^+$ LSK cells are predominantly phenotypic HSCs (i.e. CD150$^+$ CD48$^-$). In contrast, whilst the liver of naive mice contains small numbers of Fgd5$^+$ LSK cells, these are greatly reduced in infection, suggesting the liver is not a major site of HSC residency in chronic schistosomiasis. We suspect our results differ from Wijshake et al due to chronic vs. acute/fatal disease. We did detect small numbers of phenotypic MK/erythroid biased-MPP2 in infected livers (i.e. LSK CD150$^+$ CD48$^+$) but have not characterised these further. Determining the contribution of liver MPP2 (or liver resident MK) to platelet production is technically very difficult and beyond the scope of this study. Wijshake et al show that functional HSC are present in the liver after acute infection by transfer of cells into lethally irradiated recipients. We have not done this given the lack of evidence for HSC liver residency in chronic infection. Since our HSC reporter data is part of a much broader investigation into the impact of chronic schistosomiasis on HSC function (Chakraborty *et al,* in prep), we would rather not include in this submission, but show here answer the reviewers' question.

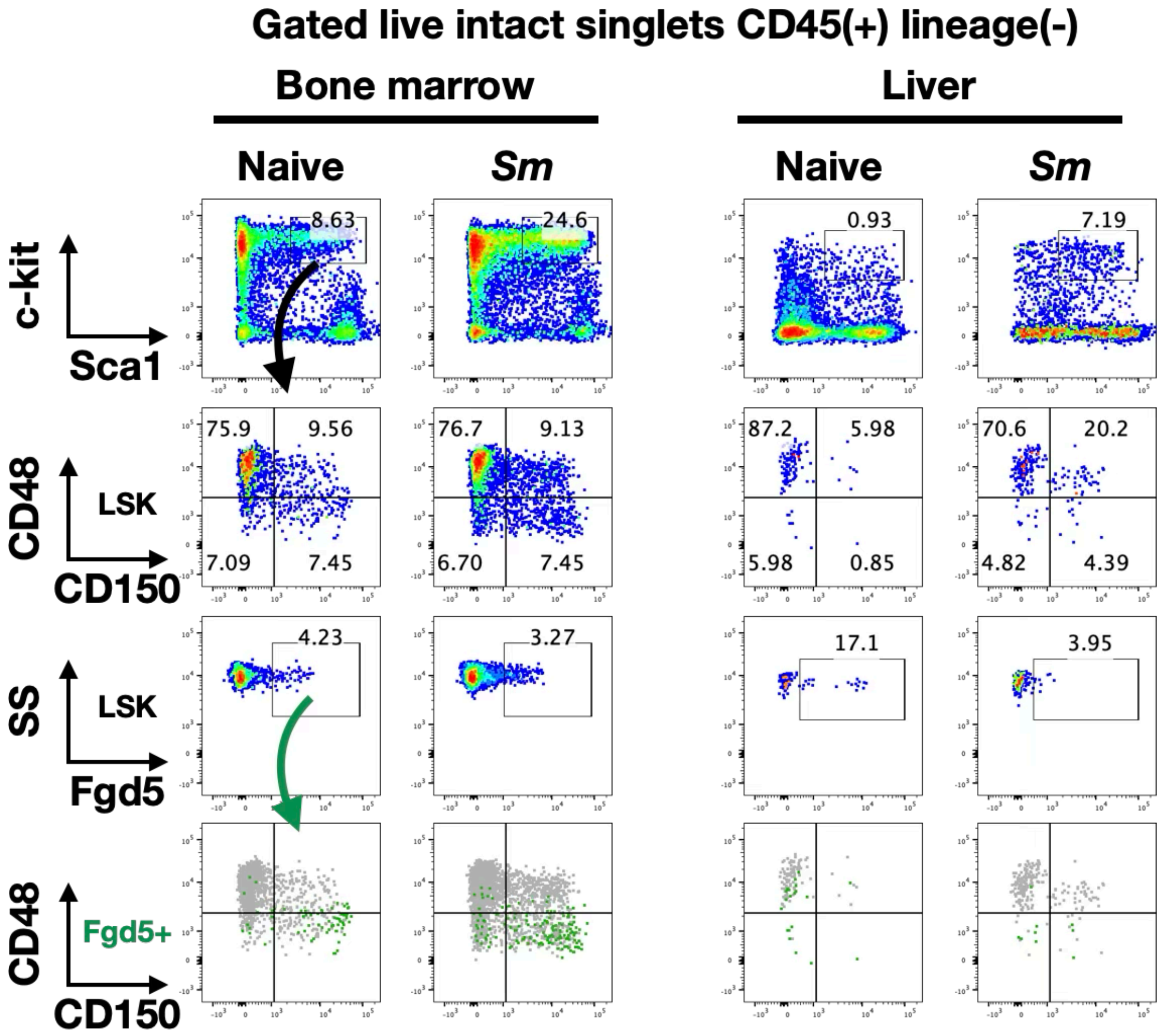

Response Figure 1. Chronic infection does not induce expansion of phenotypic HSC in the liver. Bone marrow and livers were recovered from naive and 10wk Sm-infected Fgd5 zsGreen reporter mice. Cells were stained for CD45.2, a lineage cocktail (CD11b, B220, CD19, CD3, Ly6G, F4/80, NK1.1) and other indicated markers. LSK cells were identified and SLAM marker (CD150/CD48) expression assessed. Fgd5 zsGreen expression in gated LSK cells is shown. Whilst BM LSK Fgd5^{+} cells are predominantly CD150^{+} CD48^{-} phenotypic HSC, these are not found in the liver at 10wks post-infection.

4) The authors want to study inflammatory megakaryocytes by measuring the ploidy. I recommend using one or more of the markers suggested in the original paper by Sun et al, e.g. CD53

As suggested by the reviewer, we have complemented our MK ploidy analysis by measuring MK CD53, LSP1, MYLK4 and ARNTL expression. Consistent with Sun et al, we find CD53 is expressed by a subpopulation of CD41^{+} MK but interestingly this does not change following schistosome infection. In our hands, ARNTL and MYLK4 are expressed by ~ all MK (again levels unchanged by infection). In contrast, we now show schistosome infection causes an increase in the proportion of LSP1^{+} MK

compared to naive animals, consistent with Sun *et al* showing these cells are low ploidy “immune-biased” MK. This new data is included in Fig. S2E-F.

5) What is the authors conclusion where the increased platelet clearance takes place? To prove/exclude the role of liver and spleen I suggest performing cryosections on these tissue and stain for macrophages and platelets to see how many platelets are retained in the respective organs.

Whilst we have stained spleens and liver sections for CD41 (including Fig. 2A and S6D), we find individual platelets are too small to visualise in this way, and instead cryosections show only larger MK and microthrombi (the latter are particularly prevalent after higher dose infections, [79]). This inability to see cells interacting with individual platelets using confocal motivated our *in vivo* X649-platelet labelling experiments in Fig. 4. We now comment in the Discussion that the role of the spleen in infection-induced thrombocytopenia could be clarified using splenectomised mice (but similar experiments with the liver are clearly not possible).

6) To investigate altered macrophage phenotype following infection and treatment with romiplostim or in Mpl-deficient animals the authors provide MHCII expression levels. I recommend also checking for other markers shown in figure 5 under these conditions to get a better understanding if there are changes in the macrophage phenotype.

We also measure RELMα in macrophages from romiplostim-treated mice (Fig. S6M) and MPL$^{-/-}$ animals (Fig. S7L). To provide additional comparison, we now include new experiments whereby we carry out qPCR on sorted liver macrophages from infected WT vs. MPL$^{-/-}$ mice for *Tnf*, *Il6*, *Il10*, *Cxcl12*, and *Retnla* (RELMα). This is included in Fig. S7M. Unexpectedly, this revealed macrophages from MPL$^{-/-}$ mice (with low platelet numbers) have reduced expression of *Tnf* and elevated *Il6* compared to WT macrophages (with no change in *Retnla*). This is surprising as experiments in Fig. 5 show platelet-associated macrophages also have reduced *Tnf* and elevated *Il6*. Together with our *in vivo* romiplostim and *in vitro* macrophage-platelet co-culture experiments, our interpretation of this discrepancy is that platelets are not the main drivers of these alterations in macrophage phenotype (a point we make in the Discussion).

7) Lastly, does romiplostim or Mpl knockout have an impact on the infection/inflammation itself?

We show that romiplostim does not alter disease pathology in Fig. S6H-K (liver/spleen weights, liver granulomas, egg numbers). We now include similar data for MPL$^{-/-}$ mice showing MPL deficiency does not alter infection-induced hepatosplenomegaly, granuloma size or liver egg burden (Fig. S7D-G).

Reviewer #3:

PZQ treatment doesn’t eliminate eggs within the period they examined, due to the time need to resolve granulomas. Remaining eggs in liver and or circulating SEA should be quantified as this phenomenon may be driven specifically by SEA. This could also be addressed via SEA or egg injection of mice. Does that recapitulate the platelet effects or is infection needed?

We have assessed the impact of schistosome egg injection on platelet number and size. Eggs (~5K) were injected i.p. on d0 and i.v. on d14, with bleeds carried out at d21. The initial i.p. injection induces type 2 immune responses which are then directed to the lungs by i.v. egg delivery. We find that eggs do not reduce platelet numbers, but there is a small increase in platelet size (albeit not as marked as following infection). This data is now included in Fig. S1G-H. Shortly after our original submission, another group published a very similar study (Mickael et al 2024, [39]). In that paper, egg injection causes a modest reduction in platelets as well as increased MPV (as we see) albeit at 3d post-final injection rather than our 7d. We now cite this study. It is possible multiple egg injections over a longer time frame will impact platelets to a greater extent, but carrying this out would require changes to our UK Home Office Project Licence for animal work that would further delay this submission. We hope the reviewer agrees that our experiment and that of Mickael et al show eggs alone have (minor) effects on platelets.

The IL-4 data is interesting, IL-4c injection for a few weeks would be more relevant in determining its role in the current phenotype than IL-4 exposure in culture

We have carried out an IL-4c experiment (1µg rIL-4 complexed with 5µg anti IL-4 mAb i.p. on days 0, 2, 4, 7 and 9, then assess platelet parameters at d10). In our hands, this causes a small reduction in platelet numbers as well as a moderate increase in MPV (now included in Fig. S2J-K). Notably, an earlier study (Milner et al Blood 2010) [46] which we now cite used osmotic pumps to deliver 1µg rIL-4 per hour for 3d, which caused substantial thrombocytopenia and even death. They also saw similar results with 3 injections of IL-4c over a one week period (10µg rIL-4 : 50µg anti-IL-4). Whilst Millet et al and our own experiments are relatively short term (rather than schistosome infection over many weeks), they both indicate that IL-4 signaling alone has (some) impact on *in vivo* platelet parameters.

Figure 5 the gene expression profile is interesting, but given the critical role in schistosome pathogenesis for IL-10 it would be easier to place the data in the context of the literature if IL-10 production was demonstrated. The data should at least show IL-10 relative transcript levels for each population (CD41+/-) separately within infected, uninfected control and leishmania infected individuals if IL-10 protein can't be measured.

We do compare IL-10 transcript levels between $CD41^+$ and $CD41^-$ liver macrophages from infected mice in Fig. 5F and find no difference. We could not carry out comparisons with liver macrophages from naïve mice as sorted cell recovery was already very low from infected mice (especially for $CD41^+$ cells, ~1-10K). This would be even lower in naive mice due to combination of reduced number of liver macrophages and reduced proportion that are $CD41^+$.

More minor point, IL-4 driven alternative activation of macrophages is not the same as schistosome driven (different levels of key transcripts like Relm, Arg1,etc). So the use of IL-4 to activate the macs in the culture experiment does not rule out an effect of the plate interaction on schistosome activated BMDM. Better experiment would be 24 hr SEA stimulation of 7 day BMDM with and without platelets from infected and naive mice

The reviewer makes a good point here. We carried out the suggested experiment with IL-4-activated BMDM -/+ SEA either without platelets, with platelets from naive mice, or platelets from schistosome-infected mice. The most striking finding was that SEA treatment actually reduces RELMα expression by IL-4-treated macrophages. We do not have an explanation for this but want to identify the parasite molecules responsible. We also find platelets from infected (but not naive) mice cause an additional minor reduction in RELMα. This new data is now included in Fig. S5J.

Part III – Minor Issues: Editorial and Data Presentation Modifications

Reviewer #1:
Minor point: The figure legend to figure 2 says that TPO staining is in yellow, but it is in red.

This is now corrected.

Reviewer #2:

<u>More critical minor issues</u> (e.g. study conclusions should be slightly revised if further analysis or experimentation are not performed):

1. It is stated that TPO production by hepatocytes is impaired, however this is not directly measured. The liver increases in size during schistosomiasis, and so although the area of TPO-producing cells decreases in Fig.2A this does not necessarily reflect a reduction in total TPO producing cells. The TPO brightness in the non-granulomatous liver regions seem brighter after Sm infection (fewer hepatocytes but more TPO?). Better quantification of total TPO production by hepatocytes is required to state that TPO production by hepatocytes is impaired. If this is not done the statement that TPO production by hepatocytes is reduced should be revised.

We agree with the reviewers' point here - whilst we show that granulomas formed by infiltrating immune cells are devoid of TPO-producing cells (i.e. hepatocytes), overall TPO producing capacity in the liver may be maintained because the organ increases in size. We do show that serum TPO is markedly reduced after infection (Fig. 2B), but this could also be explained by e.g. increased consumption. In addition, newly added *Tpho* qPCR data (new Fig. S2B) also does not take into account increased liver size. We have not been successful in our attempts to isolate live hepatocytes from naive and infected liver, which would allow us to quantity TPO protein/transcript on a per cell basis. As such, we have adjusted the text to state infection causes reduction in systemic TPO levels (Fig. 2B), but we avoid saying this is due to reduced production.

2. Fig. S5I - no effect observed when platelets where added to BMac. This experiment was designed to determine whether platelets directly modulate macrophage alternative activation. The experiment seems sorely lacking a positive control. Specifically the authors state that in other conditions platelets directly impact macrophage activation - could you apply these conditions to the platelets before adding to the BMacs? I worry that the BMacs do not reflect liver macrophages, with the platelets perhaps not interacting at all with the BMacs (in contradiction to your in vivo findings!). Without a positive control I do not believe you can use this experiment

to make any statement about whether platelets do or do not impact macrophage activation.

We do cite multiple studies (ref 12-18) showing platelets regulate different myeloid cells. From these, only two focus on mouse macrophages and include *in vitro* experiments. Xiang *et al* (ref 12) show LPS-stimulated mouse BMMφ express lower levels of TNFα and IL-6 following co-culture with platelets. Conversely, Barrett *et al* (ref 13) show mouse thioglycollate-elicited macrophages express elevated IL-6 and IL-1β following platelet co-culture. Given these discrepancies, we have carried out multiple experiments to test the impact of platelets on *in vitro* mouse BMMφ. In our hands, platelets do not reproducibly alter macrophage inflammatory cytokine production, as judged by *Tnf* expression (**Response Fig. 2**). As such, we do not have a reproducible positive control. One reproducible finding is that platelets modify macrophage phagocytosis, but this is part of a larger set of *in vitro* experiments we hope to publish separately, and we think this beyond the scope of this (predominantly) *in vivo* study.

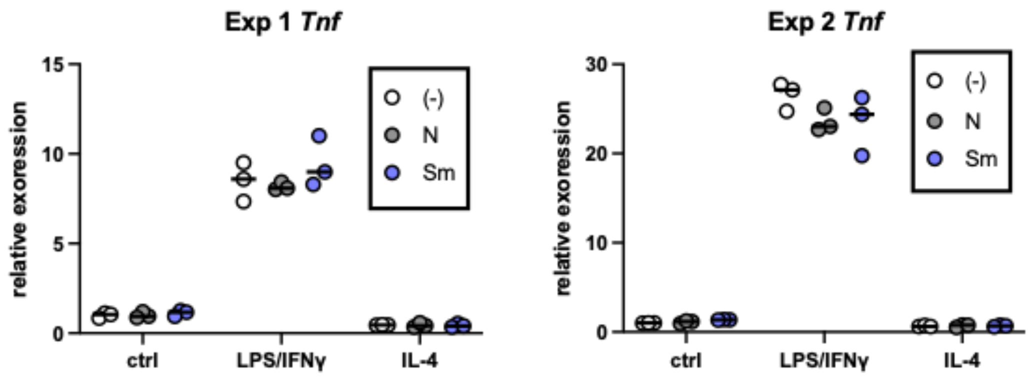

Response Figure 2. Platelets do not alter inflammatory cytokine expression by mouse BMMφ. Macrophages were cultured overnight under unpolarised control conditions or following M1 (LPS/IFNγ) or M2 (IL-4) stimulation in the absence of platelets (-) or with 100:1 platelets : cell ratio, with platelets from naive (N) or schistosome-infected (Sm) mice. *Tnf* expression was assessed by qPCR.

We address the broader point about better modeling macrophages in schistosome infection with new our data assessing RELMα in BMMφ treated -/+ SEA (Fig. S5J), as discussed in our response to Reviewer 2 above. We do agree there are likely differences between *in vivo* liver macrophages and *in vitro* studies with BMMφ. This would be greatest between liver-resident Kupffer cells and monocyte-derived macrophages (reflecting differences in ontogeny, tissue factors etc). However, liver resident macs are quickly replaced by monocyte-derived macrophages in schistosome infection (PMID: 30919955), so our use of BMMφ seems reasonable here.

<u>Actually minor issues</u>:

1. Please add references for the mouse strains used (p6, line 111-113) to allow readers to get further information on the strain and genetics if required.

We now indicate WT mice are C57BL/6J. The FcRγ chain deficient mice were produced as part of the large KO Mouse Project (KOMP,

https://www.komp.org/redirect.html) and do not have a reference beyond the code given. We have added the reference for MPL deficient mice,

2. Page 8 line 148 it states control mice received vehicle alone but the vehicle is not stated. What is the vehicle?

PBS, we have modified the text.

3. Page 14 line 290 - Figure S1B is referenced after the statement "TPO is produced primarily by hepatocytes in the liver, which is the major site of granulomatous inflammation in S. mansoni infection". Figure S1B is liver weights and so does not show TPO production and is not (save incredibly indirectly) a measure of granulomatous inflammation. Please use literature references (or some of your own more-direct data) instead.

We have rephrased the sentence to make clearer that the liver in site of inflammation ("TPO is produced primarily by hepatocytes in the liver, an organ which is a major site of inflammation in *S. mansoni* infection").

4. Figure 3C - what do the significance stars refer to? Are you comparing between groups or between timepoints within groups - please clarify.

Apologies. Significance stars were inadvertently duplicated during the production of Fig. 3C, hence orange stars appeared twice (this has been fixed). We have amended the figure legend to make clearer that significance refers to infected groups *vs* naive controls at each time point.

5. Figure 4D - what is the control column? X649 not added? Please clarify.

PBS alone, we have modified figure legend.

6. Line 440 page 20 - is Retlna an anti-inflammatory factor? Elsewhere in the paper you refer to it as an alternative (M2) or Th2 associated factor which seems more accurate to me.

We view RELMα both as a factor expressed by alternatively activated macrophages (and eosinophils) and also as an anti-inflammatory molecule. Evidence for the latter includes RELMα$^{-/-}$ animals showing increased granuloma size and inflammation following schistosome egg injection/infection (PMID: 19381262, PMID: 19349464).

7. Line 633 page 27 - the hypothesis that intestinal bleeding allows gut bacterial translocation and therefore an elevated type 1 response comes a bit out of the blue. Can you provide more evidence either from literature or your own research to support this.

We see the reviewers point and have added references to support this. We also highlight that this is a hypothesis which would need testing for MPL$^{-/-}$ mice.

8. Similarly the mentions of pseudo-thrombocytopenia seem to come a bit out of the blue. It would be helpful to include a definition of what you mean by this term. My

understanding is that it is a clinical term to refer to spurious/misdiagnosis of thrombocytopenia due to clumping of platelets either alone or with leukocytes. What you are seeing seems to be a real biological phenomenon of elevated myeloid numbers in Sm (in blood but also tissue) allowing for more platelet binding, contributing to reduced platelet numbers in blood. Is this really pseudo-thrombocytopenia?

We agree with the reviewer and have rephrased to clarify that enhanced immune cell-platelet interactions may contribute to the reduced numbers of circulating platelets (without incorrect usage of term pseudothrombocytopenia).

9. The assertion that thrombocytopenia is partially reduced upon PZQ treatment is a bit of a stretch. There is no significant difference between platelet numbers in Sm mice and Sm+PZQ mice. The assertion that thrombocytopenia is maintained after schistosomiasis infection is more in line with your data as well as perhaps even more interesting (and therapeutically relevant).

We agree with the reviewer and have replaced “partially recovers” with “does not fully recover”. The reviewer is right that the main finding is PZQ treated mice still show significantly lower platelet levels compared with uninfected animals. We were trying to strike a balance since (some) PZQ treated mice trend towards recovery, but went too far the other way.

10. A key finding is that enhanced platelet clearance in Sm is associated with increased binding of platelets to Mo/Macs. You show that this is because there are more Mo/Macs binding platelets in Sm infection (Fig 4F), but also that the percentage of Mo/Macs that bind platelets increases (Supp. Fig. S4C and D). The latter point is crucial and I believe should be in the main figures not supplement.

We considered the reviewers suggestion but would prefer to keep the CD41$^+$ cell number data in Fig. 4 and % X649$^+$ in Fig. S4. This is because combining the two can be confusing as X649$^+$ cell number reflects both % X649+ cells and total cell number (which then must also be included in main figure for explanation). Additionally, high % X649$^+$ does not always reflect high cell number, as is the case with non-classical blood monocytes (i.e. high % X649$^+$ but low X649$^+$ cell number as these monocytes are reduced on infection). We try highlight these points in the main text, but think collating all the data in Fig. 4 would make interpretation of the figure more difficult.

Reviewer #3:
Unclear why fecal egg counts for consecutive days were not included in figure 6.

Unfortunately we did not carry out longitudinal fecal egg counts in either romiplostim-treated or Mpl$^{-/-}$ mice. We add this caveat to the Results (section 3.6).

Why was relmalpha the only marker looked at? CD206/301 would be informative here in the conclusion that M2 activation is unaffected.

Whilst we did not include CD206 or CD301 in these flow panels, we routinely stain for Ym-1 when assessing alternatively activated macrophages. However, over the course of this study, we found Ym1 is not always induced in liver macrophages in schistosome infection (whereas RELMα is). This contrasts with our previous work and those of

colleagues looking at Ym1 in multiple other helminth infections (*Heligmosomoides polygyrus, Nippostrongylus brasiliensis, Litomosoides sigmodontis, Brugia malayi*), suggesting it is either a facet of schistosome infection or potentially liver M2 macrophages.

Also why are only splenic CD4 evaluated here, liver would be informative as the authors demonstrated aggregates and extramedullary hematopoiesis in the liver. The data in Figure 7 are likely the best that can be done given the lethality of infection in the absence of platelets, however, the data reenforce the need to do the culture experiments in Figure 5 in a way that actually tests schistosome infection relevant mechanisms

We include liver CD4 T cell ICC following romiplostim-treatment in Fig. S6N. We also now include the SEA macrophage culture as discussed earlier.

To summarise, we have added the following new data/panels: Fig. 2A, Fig. S1G-H, Fig. S2B, E-F, J-K, Fig. S5J, Fig. S7D-G, M. We have also moved Fig. 7A into the main paper (it was in Fig. S7 in our initial submission, but we think it should be in main paper).

We hope the reviewers agree we have addressed their concerns and comments and thank them for their time which has improved and clarified the manuscript.

Yours sincerely

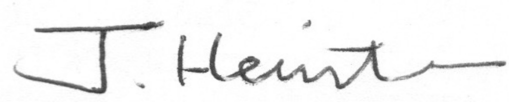

James Hewitson, PhD
On behalf of other authors
